# Learning to use a body-powered prosthesis: changes in functionality and kinematics

**DOI:** 10.1186/s12984-016-0197-7

**Published:** 2016-10-07

**Authors:** Laura H. B. Huinink, Hanneke Bouwsema, Dick H. Plettenburg, Corry K. van der Sluis, Raoul M. Bongers

**Affiliations:** 1 Center for Human Movement Sciences, University of Groningen, University Medical Center Groningen, UMCG sector F, FA 23, PO Box 196, Groningen, NL-9700 AD The Netherlands; 2 Adelante Rehabilitation Centre, Centre of Expertise in Rehabilitation and Audiology, Hoensbroek, The Netherlands; 3 Department of Rehabilitation Medicine, Research School CAPHRI, Maastricht University, Maastricht, The Netherlands; 4 Department of BioMechanical Engineering, Faculty of Mechanical, Maritime, and Materials Engineering, Delft University of Technology, Delft, The Netherlands; 5 Center for Rehabilitation, University of Groningen, University Medical Center Groningen, Groningen, The Netherlands

**Keywords:** Upper-limb prosthesis, Body-powered prosthetic hook, Prosthetic training, Proprioceptive feedback, Grip force control, Action-perception, Amputee, Kinematics, Functional performance

## Abstract

**Background:**

Little is known about action-perception learning processes underlying prosthetic skills in body-powered prosthesis users. Body-powered prostheses are controlled through a harness connected by a cable that might provide for limited proprioceptive feedback. This study aims to test transfer of training basic tasks to functional tasks and to describe the changes over time in kinematics of basic tasks of novice body-powered prosthesis users.

**Methods:**

Thirty able-bodied participants and 17 controls participated in the study, using a body-powered prosthetic simulator. Participants in the training group were divided over four groups and practiced during a 2-week-period either direct grasping, indirect grasping, fixation, or a combination of these tasks. Deformable objects with different compliances had to be manipulated while kinematic variables and grip force control were assessed. Functional performance was measured with the Southampton Hand Assessment Procedure (SHAP) prior to and after the training sessions, and after 2 weeks and 3 months retention. The control group only performed the SHAP tests.

**Results:**

All four training groups and the control group improved on the SHAP, also after a period of non-use. Type of training had a small but significant influence on the improvements of the SHAP score. On a kinematic level movement times decreased and hook closing velocities increased over time. The indirect grasping group showed significantly shorter plateau times than the other training groups. Grip force control only improved a little over training.

**Conclusions:**

Training action-perception couplings of body-powered prosthesis in basic tasks transferred to functional tasks and this lasted after a period of non-use. During training movement times decreased and the indirect grasping group showed advantages. It is advisable to start body-powered training with indirect grasping tasks but also to practice hook-object orientations.

**Electronic supplementary material:**

The online version of this article (doi:10.1186/s12984-016-0197-7) contains supplementary material, which is available to authorized users.

## Background

Body-powered prostheses are a commonly used type of prosthesis among persons with an upper-limb amputation. Strikingly there are high rates of rejection among prosthesis users, ranging between 16 and 58 % for body-powered prostheses [1]. This is due to several factors like an unattractive appearance of the prosthesis, pain and discomfort during wearing and dissatisfaction about the received preparation and training [2–4]. Earlier studies showed that training enhances functional prosthesis use, and it was suggested training would improve the acceptance of the prosthetic device as well [5–7]. Examining the processes of learning underlying the acquisition of prosthetic skills and identifying the functionally relevant aspects of learning is novel for body-powered prostheses and is the focus of the current paper. Such knowledge could be helpful in developing an evidence-based training that might solve part of the dissatisfaction with regard to functional use.

Functional use of a prosthesis is usually measured with a testing instrument that analyzes task completion times of activities of daily living (ADL). The usage of such an instrument prior to and after a training could show whether training affected the transfer of the learned skill to the performance of functional tasks with the prosthesis. Several studies show that movement times and initiation times of ADL-based tasks decrease over a short training period for body-powered prosthesis users [5, 8, 9]. Moreover, the performance of body-powered prosthesis users improves on the Box and Block Test and the Nine Hole Peg Test after a short training period [10]. Another instrument that is frequently reported and used by clinicians is the Southampton Hand Assessment Procedure (SHAP) [11]. Wright [12] considered the SHAP as having high potential in being a good outcome measure for prosthesis use. In the SHAP, abstract objects have to be grasped, such as a sphere, and tasks from daily life have to be performed such as pouring liquid in a glass and turning a door handle. These tasks have to be performed as fast and accurate as possible and the self-timed completion time was recorded. Importantly, SHAP measures at both the function and activity level of the ICF (International Classification of Functioning, disability and health), and especially on the level of functional performance in ADL tasks we wanted to establish effects of training (cf. [13]).

The studies showing improvements in functionality and task completion over time, and also studies using the SHAP [14, 15], do not reveal the processes underlying the improvements on these tasks [16]. Getting more insight in the processes underlying task improvement reveals relevant knowledge to further advance training programs for body-powered prostheses. To understand where to focus on to get at these underlying processes we have to characterize the use of body-powered prostheses. Body-powered prostheses are controlled through a cable connected at one end with the terminal device (i.e., the prosthetic hook or hand) and at the other end attached to the body. Usually the cable is connected to a harness that is wrapped around the contralateral shoulder (but see [17] for recent developments). Opening or closing of the prosthesis is done by movement of the upper arm, shoulders, and trunk depending on the type of harness and prosthesis. Depending on the design of the device, one of the actions (either opening or closing of the device) is under voluntary control while the other action follows from a spring. A defining characteristic of a body-powered prosthesis is that the movement of the device is directly related to movements of body-parts the harness is attached to and that forces produced at the terminal device produce forces to that body-part through the harness. Therefore, it is generally assumed that body-powered users can employ some proprioceptive feedback about the device on the contralateral shoulder, next to the visual feedback [18, 19]. So, learning to use a body-powered prosthesis implies learning the relation between the movements of the harness that opens/closes the prosthetic hand or hook, and the feedback it produces about the prosthesis.

Here we take the first steps to understand the learning of these new action-perception couplings. To do this we follow the same approach as Bouwsema et al. [13] had followed for myoelectric prostheses; we examine changes in kinematics of basic aspects of using a prosthesis. That is, we examined how kinematic landmarks of reaching and grasping changed in directly grasping an object and in indirectly grasping an object (i.e., handing over an object from the sound hand to the prosthetic device). Also participants used their prosthesis to fixate an object that is manipulated by the sound hand. Finally, the degree of compressibility differed between objects that had to be grasped, which allowed us to gauge changes in control of grip force over learning. Such knowledge is of particular relevance because one of the highest goals in rehabilitation for a prosthesis user is gaining an accurate grip force control [20]. The control of grip force is very difficult with a prosthesis due to a lack of feedback [21–28]. The earlier mentioned study of Bouwsema et al. [13] found that practicing indirect grasping tasks with a myoelectric hand leads to better force control during training than practicing direct grasping tasks, which was probably due to the extra proprioceptive information retrieved by the sound hand during indirect grasping. It is unknown if and to what extent these results hold for body-powered prostheses.

The aim of the current study was to reveal processes underlying the learning to use a body-powered prosthesis hook. We asked 1) whether skills in basic tasks transferred to performance of ADL tasks, and 2) how kinematics of prehension of objects changed over practice. Therefore, we used a pretest-training-posttest-retention design where the SHAP was used for testing and basic prehension tasks were practiced during training. We had the following expectations: At the level of functionality we expected the groups who trained prehension and fixating to have improved their performance in ADL tasks more than the control group that had no training. At the level of kinematics we expected that over the practice sessions the prehensile movements became faster and smoother, and that grip force control improved.

## Methods

### Participants

A training group of 30 right-handed able-bodied participants (14 males, 16 females; age 21.37 ± 1.81 years) and a control group of 17 right-handed able-bodied participants (7 males, 10 females; age 23.29 ± 1.45 years) participated. None of them had previous experience with the SHAP. Five participants had prior experience with a myoelectric prosthetic simulator. Because a body-powered prosthesis has a very different control system, this was not considered as an exclusion criterion. Participants in the training group were randomly assigned to one of four training groups: one group practiced direct grasping tasks (DG, *n* = 7), one group practiced indirect grasping tasks (IG, *n* = 8), one group practiced fixation tasks (FIX, *n* = 7) and there was one combination group, in which a combination of the three tasks were practiced (COM, *n* = 8). The study was approved by the Medical Ethical Committee (METc) of the University Medical Center Groningen (METc application NL31039.042.09) and all participants gave written informed consent before entering this study. All participants received a gift voucher after completing the experiments.

### Apparatus

**Fig. 1:**
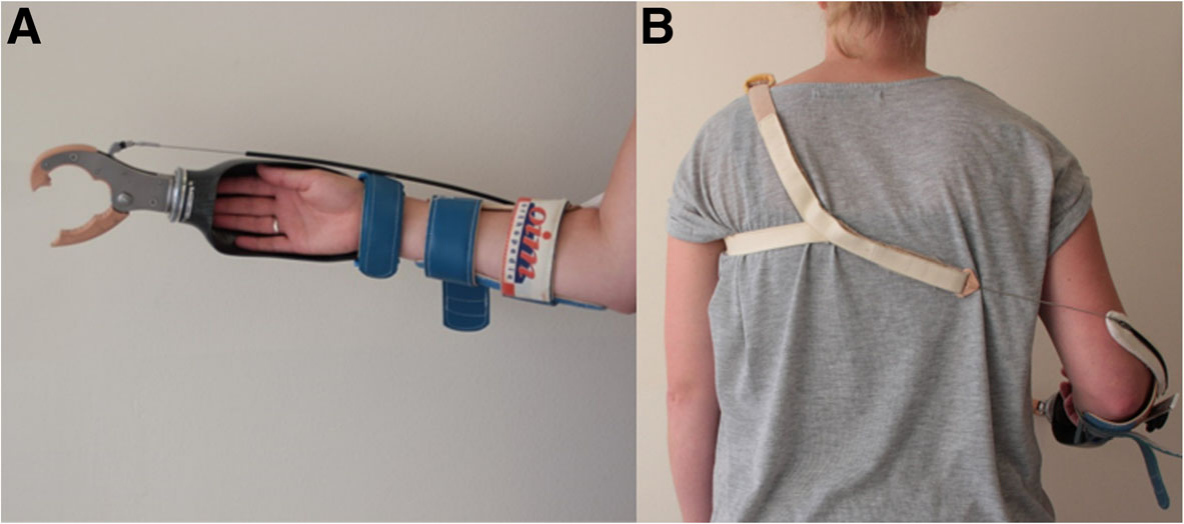
**a** The body-powered simulator. **b** The figure-of-9-harness wrapped around the contralateral shoulder

The body-powered simulator was developed to closely resemble a body-powered prosthesis for a below-elbow amputation (Fig. 1a). The simulator is controlled by a Bowden cable which is attached to a figure-of-nine shoulder harness, wrapped around the contralateral shoulder (Fig. 1b). At the other end, the cable is connected with a voluntary closing (VC) device (TRS VC Hook, Grip 3). When the cable is pulled, the hook is closed, and a spring opens the device when the cable is released. The VC hook was attached to an open cast in which the anatomical hand could be placed. Along the forearm a splint was attached to the cast, which was adjustable in length, and could be secured around the arm using a Velcro sleeve. The reason we have chosen the TRS hook is because a study of Smit & Plettenburg [29] demonstrated the TRS hook required the smallest activation force and the lowest energy dissipation.

The position of both distal ends of the hook and the objects used during the experiment were measured with two Optotrak 3020 systems (Northern Digital, Waterloo, Canada, sampling frequency 100 Hz), which recorded the positions of infrared light emitting diodes (LEDSs). Two LEDs were placed distally on the VC body-powered hook and two on each object.

To measure functional performance the SHAP [11] was used. The SHAP consists of 26 tasks, 14 ADL tasks, and 12 tasks using six abstract objects, in a lightweight and heavyweight version. The maximum opening of the body-powered TRS hook appeared to be smaller than the opening size required to grasp some of the objects used in SHAP. Therefore the smaller objects of the SHAP-C [30] were used. Standard instructions of the SHAP were given during the tests [31] however, participants were not allowed to practice the tasks beforehand, to avoid learning of the use of the prosthesis during the pretest. The participants had to self-time their tasks by pressing a button prior to and after finishing the task. These time scores were entered into the SHAP website, which transformed these scores into an overall Index of Functionality (IoF). This score represents the functionality of the hand or hook, with a score of 100 corresponding to a normal hand function.

**Fig. 2:**
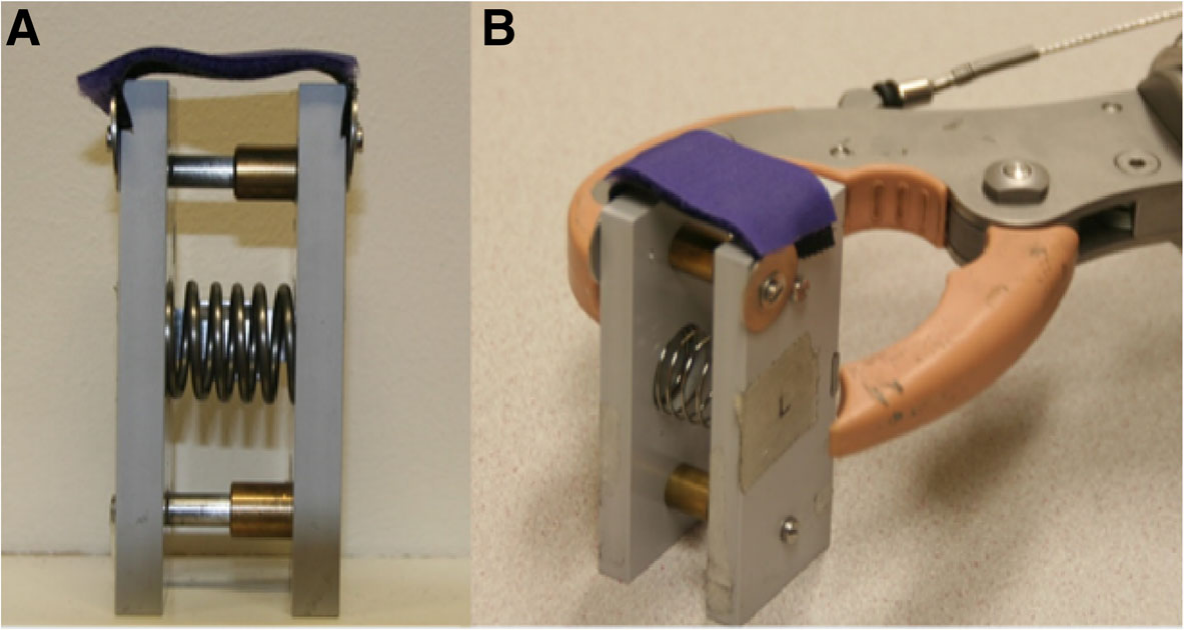
**a** A deformable object consisting of two plates with a spring in between and with a Velcro strip mounted on top. **b** A deformable object grasped with the prosthesis

During the grasping tasks three deformable objects were used (size 3.8 × 3.6 × 9 cm), each with a different resistance to deformation (see Fig. 2), and one solid object with the same measurements. The deformable objects represented objects in daily life, like a plastic cup or a carton. The objects consisted of two plates with a spring in between. The stiffness of the springs was varied to create a low-resistance object (LO, *c* = 0.83 N/mm), a moderate-resistance object (MO, *c* = 1.42 N/mm) and a high-resistance object (HO, *c* = 3.92 N/mm). On top of each object a Velcro strip was mounted, which had to be pulled off during the task. This represents manipulation of objects during daily living, like opening a carton.

### Procedure and design

#### Functional test

**Fig. 3:**
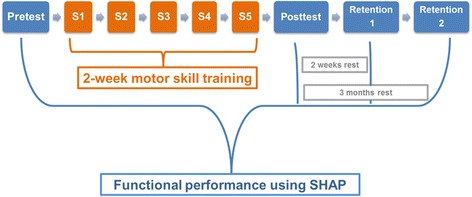
Experimental set-up. S = Session

The SHAP test was performed prior to the training sessions, to determine baseline performance with the prosthetic simulator. Directly after the last training session the SHAP had to be performed again during the posttest, to determine if the participants improved their skills on the tests. In order to determine if there was an effect of training over a longer period, a retention test was administered after 2 weeks (RT1) and after 3 months (RT2). Figure 3 shows the experimental set-up. Instructions were given before the start of the task. The control group only performed the SHAP tests, and did not practice during the training sessions of the training group.

#### Training sessions

The five training sessions of the training groups were distributed over a period of 2 weeks, to simulate a rehabilitation treatment. At the start of each training session the prosthesis was fitted.

The group performing DG tasks had to pick up one of the four objects that was placed in front of them with their prosthetic hook and subsequently had to manipulate the object by pulling off the Velcro strip with their sound hand and place the object back on the table with the prosthetic hook. The start position of the object was 45 cm from the edge of the table, whereas the start position of the prosthetic hook was at 15 cm from the edge, all in line with their right shoulder. During IG the object was initially situated in the sound hand. The starting positions of both the sound hand and the hook were at 25 cm from the edge of the table opposite to each other in the frontal plane, with 30 cm distance between the sound hand and the prosthetic hook. At the start of each trial the object was picked up with the sound hand and handed over to the prosthetic hook in midair. Subsequently the object was manipulated again by pulling off the Velcro strip with the sound hand and placed back on the starting position of the prosthetic hook. The midline of the body was aligned with the middle between the sound hand and the hook. Participants in both groups were instructed to perform the tasks as quickly and as accurately as possible, while trying not to deform the objects.

During FIX four different tasks were executed. Participants had to fixate 1) a case with a flat design and zipper located at one side on top of the case, while unzipping and zipping the case with the sound hand; 2) a ruler on top of two dots, placed 20 cm horizontally from each other, with the prosthesis, while drawing a straight line between the dots with a pencil held in the sound hand; 3) a sharpener to sharpen a pencil by turning the handle of the sharpener three times with the sound hand; and 4) a piece of cloth to unbutton three buttons. The to be fixated objects were placed 25 cm from the edge of the force plate, aligned with the body midline. Participants were instructed to fixate the object with the prosthesis as still as possible while performing the task.

In order to capture the natural development of changes in movement over time, no further instructions were given for all types of tasks (DG, IG, and FIX). Participants were not allowed to practice with the objects beforehand. Moreover, they were aware about the differences in the stiffness of the spring in the object because they were informed before each trial about the stiffness and through marks on the object. Each session consisted of 60 trials. With each of the four objects (DG and IG) and the four fixation tasks (FIX), 15 trials were performed in a random order, resulting in 60 trials per session for the DG, IG and FIX group. The COM group performed five trials per object and per task (DG, IG and FIX), resulting in 20 trials per task and thus 60 trials per session, with a randomized order of tasks (resulting in a blocked-repeated structure).

### Data analysis

#### Analysis of functional tests

A repeated measures ANOVA was executed on the IoF scores of the SHAP data. To test the differences in performance over multiple sessions, test (pretest, posttest, RT1 and RT2) was used as within-subject factor, and group (DG, IG, COM, FIX, control) as between-subject factor. In order to see whether the baseline performance of all four training groups and the control group was equal, an one-way ANOVA was performed with group (DG, IG, COM, FIX, control) as between-subject factor and the IoF score of the pretest as dependent variable.

#### Analysis of the training sessions

To determine the onset and the end in the movements executed during the grasping tasks, the Multiple Sources of Information method was used [32], which was implemented in custom written Matlab programs. For the transport phase, reach time was calculated. For the grasping phase plateau time, hook closing time, and peak velocity of hook closing were determined. The analyzed variables were defined in the same manner as in Bouwsema et al. [13], except for the plateau phase for which the onset was determined at the start of the reach, instead of the moment the hook aperture is at its maximum. This was because the hook in the current study already started in its maximum aperture position due to the characteristics of the VC hook, instead of starting with a closed hand as in the study of Bouwsema et al. [13]. The hook aperture was defined by the 3D distance between the markers on the hook representing the thumb and index finger. Likewise, the 3D distance between the two markers on the objects were determined in order to calculate the compression and the grasping force applied to the object while grasping and during manipulation.

Data were processed using Matlab (The Mathworks Inc, Ma, USA). In case markers were obscured so that one or more of the above mentioned variables could not be determined, trials were rejected. On each of the dependent variables for the grasping tasks (reach time, plateau time, hook closing time, peak velocity of hook closing, compression while grasping and compression during manipulation) repeated measures ANOVA’s were used with session (session 1 to session 5) and object (solid, HO, MO, and LO) as within-subject factors and group (DG, IG, COM) as between-subject factor. To check the normality distribution of the data, residuals of the ANOVA were analyzed using the Shapiro-Wilk test and a Q-Q plot. When sphericity was violated, the degrees of freedom were adjusted with the Greenhouse-Geisser correction. An α of 0.05 was used. On the significant main effects post hoc tests were performed using Bonferroni corrections. To calculate the effect sizes, the general eta squared was used [33], and were interpreted according to Cohen’s recommendation [34] regarding to 0.02 for a small effect, 0.13 for a medium effect, and 0.26 for a large effect. Only the effects of 0.02 and larger will be discussed in the results.

## Results

### Functional performance

**Fig. 4:**
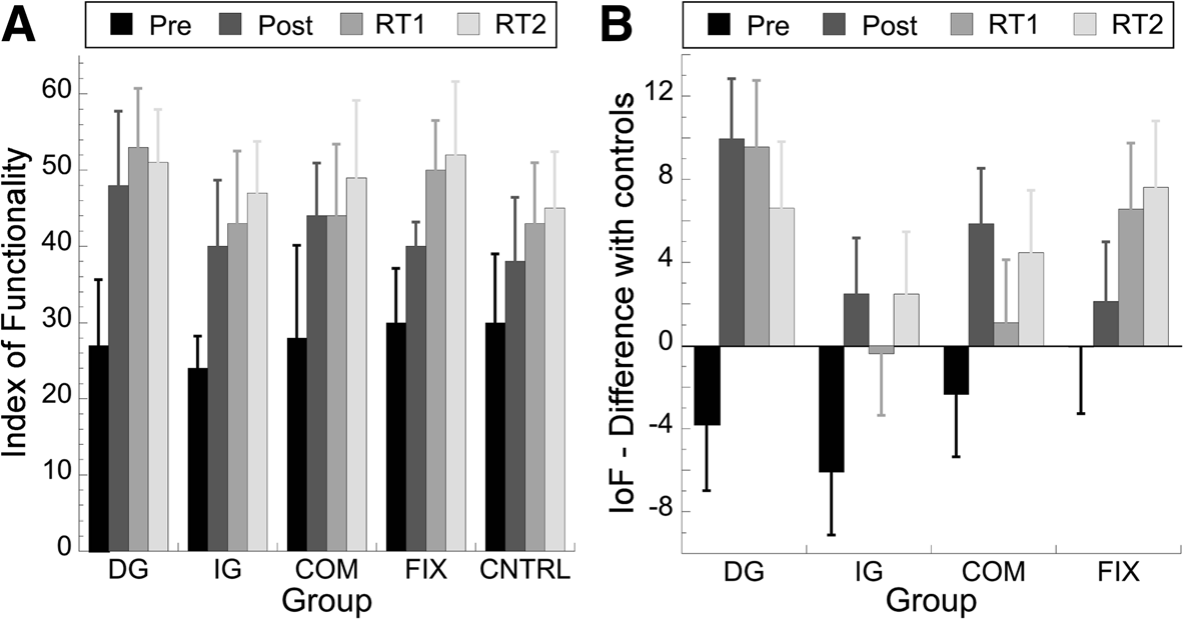
Index of Functionality scores of the SHAP. **a** Means (+/−SD) of the IoF scores are shown for the four training groups (FIX, COM, IG, DG) and the control group on pretest, posttest, retention test after 2 weeks (RT1) and retention test after 3 months (RT2). Higher scores indicate a better performance. **b** The difference between the IoF scores of the control group and each of the experimental group is plotted for each of the experimental groups at each measurement moment

In Fig. 4a, the IoF scores of the SHAP are presented for each group on each test to give an indication about progress made over the tests (Additional files 1 and 2). The SHAP data were normally distributed. A one-way ANOVA on the IoF scores of the pretest showed that baseline performance of all groups was equal (F _(4,42)_ = 0.83; *p* > 0.05). A repeated measures ANOVA on the IoF-scores demonstrated a main effect of test, in that the four training groups and control group improved on IoF-scores on the posttest, RT1 and RT2 compared with the pretest and also improved from posttest to RT1 and RT2 (*p*’s < .001 in pairwise comparison) (Table 1). Moreover, we found a significant interaction of test*group (F _(12, 126)_ = 3.206; *p* < 0.01; η_*G*_^2^ = .08), see Fig. 4a. As shown in Fig. 4a, the control group also improved over the tests. To further examine this interaction we plotted in Fig. 4b the difference in IoF score between the control group and each of the experimental groups. What can be seen in Fig. 4b is that the DG group showed the largest improvement compared to the controls after the training and in the first retention. For the IG and the COM group the improvement after the training and in the retention follows the same pattern. Finally, the FIX group showed a steady improvement compared to the control group over the posttest and the retention tests.

**Table 1:** Significant main effect of IoF scores on the SHAP of training and control groups

Dependent variable	Within/between subject factor		Mean (SE)	95 % CI lower-upper	F	*p*	η_*G*_^2^ ^a^
*IoF-scores*	Test	Pre	28.78 (1.31)	26.15–31.40	110.33	.00	.41
		Post	40.41 (1.22)	37.95–42.87			
		RT1	44.98 (1.31)	42.34–47.62			
		RT2	47.12 (1.21)	44.67–49.56			

*SE* standard error of the mean, 95 % *CI* lower-upper 95 % Confidence Interval, Lower bound and Upper bound, *Pre* Pretest, *Post* Post test, *RT1* 2-weeks retention test, *RT2* 3-months retention test

^a^Significant main effects shown for effect sizes ≥ 0.02

### Training sessions

**Fig. 5:**
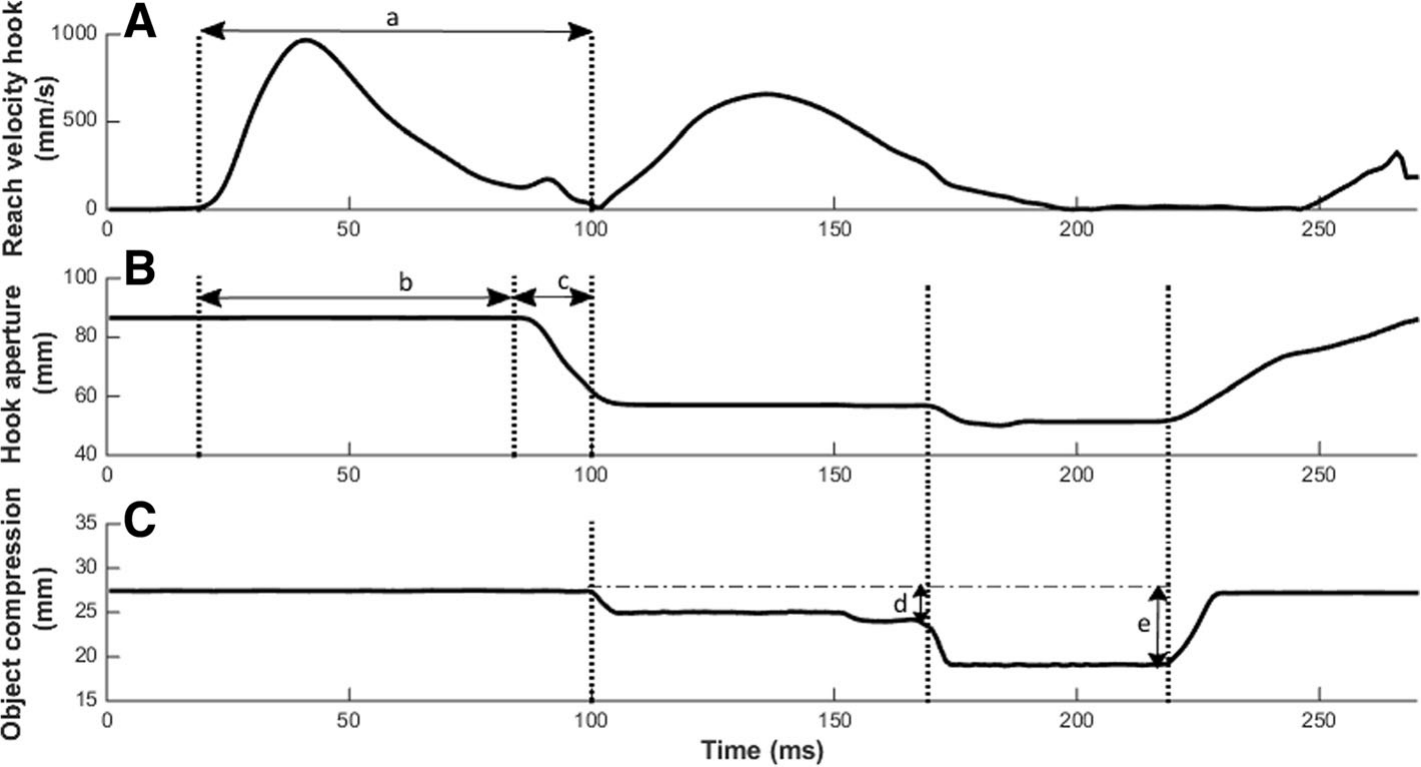
Example of a direct grasping trial with a low-resistance object. **a** Reach velocity of the hook, **b** hook aperture and, **c** the deformation of the object are plotted against the time. Several kinematic variables are represented by *a* = Reach time, *b* = Plateau time, *c* = Hook closing time, *d* = Compression at moment of grasp, *e* = Compression during manipulation

Figure 5 shows the typical actions of a body-powered hook during a direct grasping trial. The reach velocity of the hook shows an asymmetric velocity profile with a longer tail in the deceleration phase. Due to the characteristics of the VC hook, the aperture is at its maximum at the start of the trial. The plateau phase begins at the same time the hook velocity increases and ends when the hook is near the object and starts to close. During the hook closing phase the object is picked up and compression of the object starts. Compression can be divided into two phases: compression when the object is initially grasped and compression while the Velcro strip is pulled off from the object (i.e., during manipulation of the object) (Additional files 3 and 4).

All kinematic data were normally distributed. We incidentally found that the Shapiro-Wilk test was significant. Each dependent variable had 20 conditions (5 sessions, four objects), for which the hook closing time violated three out of 20 conditions, and the compression while grasping and the compression during manipulation violated one out of 20 conditions. Given the low number of occurrence on non-normality and the high robustness of ANOVA against violations of normality, we decided to continue with the ANOVA analyses. Significant main effects of each dependent variable can be found in Table 2. Over the five training sessions, body-powered prosthesis users showed a decrease in reach time, where pairwise comparisons (*p* < .001) revealed this decrease was mainly shown over the first three training sessions. There was also a main effect of group that showed the IG group had significantly shorter reach times than the DG group (*p* < .03).

**Table 2:** Significant main effects^a^ in the training sessions with an effect size ≥ 0.02

Dependent variable	Within/between subject factor		Mean (SE)	95 % CI lower-upper	F	*p*	η_*G*_^2^
Reach time (s)	Session	1	0.96 (.04)	0.85–1.08	11.63	.00	.06
		2	0.87 (.03)	0.77–0.96			
		3	0.85 (.03)	0.76–0.95			
		4	0.85 (.04)	0.75–0.95			
		5	0.85 (.04)	0.74–0.95			
	Group	COM	0.89 (.06)	0.73–1.05	4.27	.03	.26
		IG	0.75 (.06)	0.59–0.91			
		DG	0.99 (.06)	0.82–1.16			
Plateau time (s)	Group	COM	0.49 (.03)	0.41–0.57	16.71	.00	.41
		IG	0.35 (.03)	0.27–0.42			
		DG	0.57 (.03)	0.49–0.66			
Hook closing time (s)	Session	1	0.86 (.06)	0.70–1.02	20.36	.00	.20
		2	0.69 (.04)	0.59–0.79			
		3	0.65 (.03)	0.56–0.75			
		4	0.61 (.03)	0.53–0.70			
		5	0.56 (.04)	0.45–0.68			
Peak velocity hook closing (mm/s)	Session	1	134.68 (8.29)	111.10–158.26	5.19	.007	.04
		2	145.19 (10.60)	115.04–175.34			
		3	143.83 (8.50)	119.66–168.00			
		4	158.27 (11.09)	126.71–189.83			
		5	163.21 (13.32)	125.30–201.12			
	Object	Solid	157.06 (10.20)	128.04–186.08	14.43	.00	.12
		HO	151.27 (9.38)	124.58–177.96			
		MO	145.50 (9.81)	117.60–173.40			
		LO	142.32 (9.01)	116.69–167.95			
Compression when grasping (mm)	Object	HO	0.55 (.04)	0.44–0.66	402.65	.00	.83
		MO	2.47 (.11)	2.15–2.80			
		LO	5.02 (.19)	4.48–5.55			
Compression during manipulation (mm)	Object	HO	0.93 (.07)	0.74–1.12	591.65	.00	.91
		MO	5.34 (.18)	4.84–5.84			
		LO	7.30 (.16)	6.84–7.75			
Force when grasping (N)	Group	COM	3.63 (.19)	3.24–4.03	4.21	.03	.09
		IG	2.86 (.19)	2.46–3.25			
		DG	3.33 (.20)	2.90–3.76			
	Object	HO	2.14 (.15)	1.72–2.57	61.60	.00	.39
		MO	3.51 (.16)	3.05–3.97			
		LO	4.17 (.16)	3.72–4.61			
Force during manipulation (N)	Object	HO	3.66 (.26)	2.92–4.39	100.96	.00	.59
		MO	7.58 (.25)	6.88–8.29			
		LO	6.06 (.13)	5.68–6.44			

*SE* standard error of the mean, 95% *CI* lower-upper 95% Confidence Interval, Lower bound and Upper bound, *s* second, *mm* millimeter, *COM* combination group, *IG* indirect grasping group, *DG* direct grasping group, *Solid* solid object, *HO* high-resistance object, *MO* moderate-resistance object, *LO* low-resistance object

^a^A main effect of object shows the means per object over all sessions and all groups; in case of a main effect of session the means per session over all objects and all groups are shown, whereas a main effect of group shows the means per group over all sessions and all objects

The plateau time did not decrease over sessions, though a large main effect of group for the plateau time (i.e., the time in which the hook was fully open) demonstrated that the IG group had shorter plateau times than the COM and DG group (*p* < .01 in pairwise comparisons).

Hook closing time decreased over the training sessions, where pairwise comparisons (*p* < .01) revealed it did not show a clear leveling-off during the sessions. A main effect for the peak velocity of hook closing revealed that the peak velocity significantly increased over the training sessions, indicating that the hook closed with a higher speed around the object. A main effect of object demonstrated that peak closing velocity was larger when object rigidity was higher (*p* < .01 in pairwise comparisons).

For compression at the time of initial grasp and compression during manipulation a large main effect of object was found, which showed that the extent of compression was inversely related to the resistance of the object. The compression did not change over the sessions.

A large main effect of group was found for the grasp force when grasping the object, which showed that the IG group required less force than the COM group in order to pick up an object (*p* < 0.05 in pairwise comparisons). In line with the compression data, large main effects of object were also found for the grip force when grasping the object and the grip force during manipulation, indicating that the amount of force applied to the object is inversely related to the resistance of the object (Table 2).

## Discussion

The question of whether the skills acquired during practicing basic tasks with a body-powered prosthesis transfer to ADL tasks, should be answered with a pre-cautious yes. That is, we found a small but significant interaction between the type of training that participants followed and the pattern in improvements on functionality (i.e. SHAP) after the training and in retention compared to the control group. The group that had trained direct grasping showed the biggest improvement in functionality after the training. Therefore, we think that training basic tasks helps in performing ADL tasks but the relation between type of training and performance on ADL tasks, i.e. the intertask-transfer, is subtle and deserves more study. We will return to this point later. Second, we showed that over practicing the prehensile movements became faster, in particular the hook closing time. The IG group had a shorter plateau time of the grasp than the DG and COM group, but probably this is due to the contribution of the sound hand to the task in that group. As expected the object compressibility primarily affected compression and force control; the more rigid the object, the less it was compressed. Interestingly, the IG group used less force than the COM group while picking up the object, suggesting that more training with indirect grasping benefits force control of the body-powered prosthesis. Finally, during the experiments we noticed body-powered prosthesis users had difficulties continuously applying force to objects, which was due to the characteristics of the VC hook.

### Improvements in functional performance

On the functional tests, performed before and after the training, we found differences in performance depending on the type of training. Note that this was a weak effect so its interpretation needs to be done with caution. As expected [cf 15], we found that the control group improved on the SHAP over repetitions (Fig. 4a). This implies that just handling the body-powered prosthesis already leads to better performance in ADL tasks, independent on the specifics of the training that has been followed. This finding is new for body-powered prostheses. Interestingly, Fig. 4b indicated that it was not just the performance after the training but it was the pattern of change over performing repetitions of SHAP compared to the control group, which differed between the training groups. The fact that the transfer from training to the SHAP was weak might stem from the difference in the skills trained during practice sessions and the skills that were actually tested in the SHAP. Interestingly, however, after training the DG group scored highest on the SHAP and this group trained most in orienting the hook toward the object that had to be grasped. This aspect is of primary relevance in the tasks that have to be performed in the SHAP. This could explain that the IG group seemed to be slower on the SHAP than the other training groups, because the IG group (i.e. those who had to hand over an object from the sound hand to the prosthetic hook) was less familiar with hook-object positioning. On the other hand, during the training period participants mainly focused on grasping compressible objects while applying the right amount of grip force during manipulation, whereas during the SHAP the focus was mainly on grasping solid objects using different orientations of the hook, with exception of the two tasks that also required force control (pouring water from a carton and moving an empty tin can). Moreover, it should be noted that the FIX group also improved on the SHAP even though they did not actively practice prehension with the prosthetic simulator. But be aware that the SHAP also contains tasks in which objects need to be fixated with the prosthesis and that these bimanual tasks have to be coordinated with the manipulative actions of the natural hand.

Finally, in another study [35] higher IoF scores were found for body-powered prostheses with a VC device than we found with our device (55.4 vs. 41.7 in the current study). Though, participants in Berning’s [35] study were allowed to practice the SHAP tasks beforehand, which might have resulted in higher IoF scores [cf 14, 15].

### Improvements over training sessions

During the training sessions a learning effect was detected in body-powered prosthesis users, which resulted in faster movement times and an increase of peak velocity of hook closing (resulting in shorter hook closing times) over the sessions. This fine-tuning of closing the hook exhibits that over time it is learned which perceptual variables inform about the action that has to be performed. In other words, over learning the action-perception couplings are calibrated to the specifics of the task of using the body-powered prosthesis [36, 37]. However, object compression did not show a clear decrease over time, which might indicate that it takes more time to detect the relevant perceptual information for the fine control of applying the right amount of grip force to an object.

We found also differences between groups. The IG group showed a significantly shorter plateau time than the DG and COM group. These differences between the IG group and the other groups might be due to the characteristics of the IG task; the IG group had the advantage that the object could be positioned in the most favorable way in the prosthesis hook, while the DG group had to position the hook with respect to the object. The faster movement times in the IG task might also be explained by the fact that during IG the sound hand and the prosthetic hook move towards each other, causing the object to reach the prosthetic hook earlier compared with DG, where only the prosthetic hook moves towards the object. The average traveled distance of the object before it reached the prosthetic hook during IG was approximately 12 cm, which is already one third of the distance. Another explanation for the shorter plateau times might be that (proprioceptive) information from the sound hand about object properties could be used for controlling the prosthesis hook opening. We had expected this information to be less valuable than the proprioceptive information provided by the cable control. However, the opposite seemed to be the case, that is, if information from the cable provided all the information required, than we would not expect a difference between IG, DG, and COM. This is not what we found; the IG group differed from the other groups suggesting that the proprioceptive information provided through the harness about the prosthesis did not add to the performance of the DG and COM group that much that the performance of these groups equaled the performance of the IG group. Therefore, the information about the object retrieved through the sound hand might have benefitted the IG group. Moreover, the findings on grip force control showed that the IG group produced the least force during grasping the object. This suggested that the information from the sound hand in the IG task might have been more useful than the proprioceptive information produced by the harness. The limited added value of feedback from the harness might be caused by the high energy dissipation and activation forces required to operate the body-powered prosthesis [38]. Together these findings show that substantial improvements of the control mechanisms is required before the user can benefit from the richness in the action-related sensory information that is provided by the cable.

### Influence of different terminal devices

It must be considered that the results in the current study cannot be completely generalized to other types of body-powered terminal devices due to the differences between the characteristics of a body-powered hand or hook, and between VC and VO devices. Smit & Plettenburg [29] and Smit et al. [38] demonstrated that activation forces of hooks are lower than for hands in both VC and VO devices, and that not every device showed the same energy dissipation. These findings might have an influence on the grip force control, as well as that the employed proprioceptive feedback could be limited due to these high forces. A study of Haverkate et al. [10] demonstrated differences in the functional performance of different hooks. They revealed that the Hosmer hook (VO), which was also used in the study of Berning [35], scored better on the Box and Block test and on the Nine-Hole Peg Test than the TRS hook, which was used in the current study. This finding might as well explain the difference found in IoF scores between the Hosmer hook (VC) in Berning’s study [35] and the TRS hook in our study. It could be that the different shapes of the hooks influenced these scores as well.

Above all there is an intuitive control difference between VC and VO devices. VO devices are able to lock the cable while holding an object, which might as well have an influence on the evolvement of skills over learning. Thereby it has been shown that participants with a VC hook were approximately 1.3 s faster than participants using a VO hook, while performing the SHAP test [35]. In that study it was also shown that for different types of tasks, different devices were preferred. For future research it is therefore important to take into account the influences of the different characteristics of terminal devices on movement outcomes and functional performance.

### Body-powered and myoelectric prostheses

In the current study we used the same design as Bouwsema et al. [13] did with a myoelectric prosthetic hand. We had expected substantial differences between our findings and those of Bouwsema et al because of the differences in control of the body-powered hook and the myoelectric hand. Note that myoelectric prosthesis users have to learn to map the amount of muscle contractions to a certain hand opening velocity of the prosthesis. Obviously, actions can be performed faster with a body-powered prosthesis than with a myoelectric hand controlled by a motor. Moreover, the shape of the terminal device is different which implies that objects of different sizes can be grasped with the two devices and that the devices differ in the objects on which the grip is sound. Interestingly, we found an effect of group on the produced force when grasping whereas with the myoelectric prosthesis no difference between training groups was found on the compression and the produced force. It might be that these differences stem from the direct control via the cable that also gives some information about the exerted forces. As explained earlier we believe that our results show that this information is limited, therefore, our findings do not substantiate that the current body-powered prosthesis provide more extended physiological proprioception [19] than a myoelectric device.

### Evidence-based training

To date no evidence was provided in the literature with regard to the build up of a training program for novice body-powered prosthesis users. Our findings suggest that the use of the sound hand in presenting objects to the prosthesis, and probably the information of the object perceived through the sound hand, is beneficial for body-powered prosthesis use. Therefore, we suggest starting with IG tasks in training. In order to get more familiar with orientation of the hook towards the object, it is also important to practice DG tasks. This was supported by the results of the COM group in the current study who performed equally to and even showed a tendency of better performance than the DG group. However, these are just the first steps. We have shown that the proprioceptive information about the hook is limited. This requires on the one hand more technological advancements to improve the design of the hand and cable mechanism [39], as well as training to improve the perception of the produced force [40–42].

Future research should also investigate how grip force control can be improved and what the effects of augmented feedback are on performance. A study of Ninu et al. [26] already showed that providing augmented (vibrotactile) feedback about the hand closing velocity of myoelectric prosthesis users resulted in a better grip force control for objects with a lower resistance. It might be that providing feedback on the hook closing velocity of body-powered prosthesis users increases performance as well. To improve grip force control, it is important to keep in mind the difference in characteristics of terminal devices.

### Study limitations

It should be noted that in the current study we included able-bodied participants using a prosthetic simulator, instead of amputee patients. However the number of novice amputee patients without any experience with a prosthesis is very limited, while we could include more participants by using able-bodied participants to improve the power of the study. With regard to the muscles used to operate the prosthesis, for body-powered prosthesis users the muscles of the contralateral shoulder are not expected to be affected by the amputation. Note that the results on the Box and Block test of a study using a body-powered simulator [10] were comparable with the results of an amputee patient using a body-powered prosthesis [43]. Therefore we expect that our results with able-bodied participants can be used to develop training for patients using a body-powered prostheses.

In the current study we measured only right-handed participants, which might not completely generalize the results to left-handed participants. A review of Mutha et al. [44] discusses the influence of differences in handedness for motor control. They suggest the left hemisphere is specialized for predictive control whereas the right hemisphere is specialized for impedance control, which might have consequences for the development of training programs for amputees. Therefore, it might be that our recommendations are not applicable to left-handed people.

Finally, we chose the SHAP for testing the improvement in functionality following training. This is because SHAP contains a wide range of tasks used in ADL. We deliberately performed a training with basic tasks to examine the extent to which the developed action-perception couplings in these basic tasks over training transferred to ADL performance. We expected this transfer because the tasks of the SHAP are made up out of reaching and grasping towards objects and fixating objects with the prosthesis. However, we found only a weak transfer, indicating that the action-perception couplings in the basic tasks differ from those in the ADL tasks. So we would like to argue that initially the choice for the SHAP, or the type of training for that matter, could be substantiated but that taking the current findings into account, the choice for using SHAP as a test and basic tasks in a training might be adjusted for future studies.

## Conclusions

Functional performance on the SHAP and action-perception learning processes of novice body-powered prosthesis users were examined over time while performing goal-directed tasks. The training of the basic tasks only weakly transferred to functional performance in the SHAP. The results clearly showed action-perception couplings get calibrated during a 2-week training period and seem to last after a period of non-use. Grip force control only improved a little over the sessions and probably takes a longer time to learn, and might be optimized by improvements in design of the device. Implications for a body-powered training program would be to start training with IG tasks but also to practice hook-object orientation.

## Additional files

Additional file 1: Read me. (TXT 17 kb)
Additional file 2: Data IoF scores. (TXT 951 bytes)
Additional file 3: Data Kinematics. (TXT 19 kb)
Additional file 4: Data Compression/Force objects. (DOCX 47 kb)

## Abbreviations

ADL: Activities of daily living
ANOVA: Analysis of variance
BP: Body-powered prosthesis
COM: Combination group
DG: Direct grasping group
FIX: Fixating group
HO: High-resistance object
IG: Indirect grasping group
IoF: Index of Functionality
LED: Light Emitting Diode
LO: Low-resistance object
METc: Medical Ethical Committee
MO: Moderate-resistance object
RT1: Retention Test after 2 weeks
RT2: Retention Test after 3 months
SHAP: Southampton Hand Assessment Procedure
VC: Voluntary closing
VO: Voluntary opening
