# Supplementary material for: Learning to use a body-powered prosthesis: changes in functionality and kinematics
Source: J Neuroeng Rehabil. 2016 Oct 7;13:90. doi: 10.1186/s12984-016-0197-7 (PMC5054596; doi:10.1186/s12984-016-0197-7)
Supplement: Additional file 4: — Data Compression/Force objects. (DOCX 47 kb) [file 12984_2016_197_MOESM4_ESM.docx]

In the attached files the dependent variables can be found that are used in the ANOVAs of the paper. The files are the files as we have used them in SPSS.

The name of the dependent variable is followed by ‘_S1 … _S5’ indicating the session which is recorded. This is followed by ‘_Solid … _LO’ indicating the compressibility of the object.

Suppl_LearningBodyPowered_IoFscores

IoF scores

Suppl_LearningBodyPowered_kinematics

Reach Time

Plateau Time

Hook Closing Time

Peak Velocity Hook Closing

Suppl_LearningBodyPowered_CompressForce

Compression when grasping

Force when grasping

Compression when manipulating

Force when manipulating
